# Whether it is safe to start anticoagulation after intracranial hemorrhage within 2 weeks: A systematic review and meta‐analysis

**DOI:** 10.1002/ibra.12060

**Published:** 2022-08-19

**Authors:** Xue‐Yan Huang, Jun‐Yan Zhang, Chang‐Yin Yu

**Affiliations:** ^1^ Department of Neurology Affiliated Hospital of Zunyi Medical University Zunyi Guizhou China; ^2^ Department of Anesthesiology Southwest Medical University Luzhou China

**Keywords:** anticoagulation, atrial fibrillation, intracranial hemorrhage, ischemic stroke

## Abstract

Whether restarting anticoagulation (RA) treatment after intracranial hemorrhage (ICH) is still controversial. We performed a systematic review and meta‐analysis to summarize the relationship between anticoagulation after ICH with the recurrence of hemorrhagic events, ischemic events, and long‐term mortality. Medline, Embase, and the Cochrane Central Register of Controlled Trials, from inception to November 2020. We searched the published medical literature to ensure cohort studies involving ICH associated with anticoagulation in adults. Primary outcomes were long‐term mortality, hemorrhagic events, and ischemic events (myocardial infarction, pulmonary embolism, ischemic stroke, or systemic embolization). We concluded seven retrospective cohorts, including 1876 intracranial hemorrhage patients with indications of anticoagulation. The ratio of the anticoagulant restart was 35.3% (664n). RA was associated with a significantly lower incidence of recurrent ischemic events (pooled odds ratio [OR] 0.29, 95% confidence interval [CI] 0.19% to 0.45%, *p* = 0.97) and death events (pooled OR 0.56, 95% CI 0.40%–0.79%, *p* = 0.27). There is no evidence that early recovery of anticoagulation (within 2 weeks or 1 month) is associated with the occurrence of hemorrhagic events (within 2 weeks: pooled OR 0.80, 95% CI 0.3–2.12, *p* = 0.52 vs. within 1 month: pooled OR 1.14, 95% CI 0.77–1.68, *p* = 0.82). Based on these, recovery of anticoagulation after ICH is beneficial for long‐term mortality and recurrence of ischemic events. The meta‐analysis showed a resumption of oral anticoagulation within 2 weeks or 1 month in patients who had a cerebral hemorrhage was beneficial and did not increase the risk of hemorrhagic events and reduced the occurrence of ischemic and fatal endpoint events.

## Graphical abstract

The resumption of oral anticoagulation within 2 weeks or 1 month in patients who had a cerebral hemorrhage was beneficial and did not increase the risk of hemorrhagic events and reduced the occurrence of ischemic and fatal endpoint events.

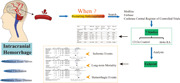

## 1 INTRODUCTION

At present, the number of ischemic stroke patients caused by atrial fibrillation (AF) or mechanical heart valves is increasing year by year. Oral anticoagulant is needed to prevent systemic embolism or cardiogenic stroke. With the aging process, patients with AF also show an increasing trend year by year. The Global Burden of Disease Project estimates that the global prevalence of AF in 2016 was approximately 46.3 million people.^1^ The prevalence of AF in Asian and Chinese populations ranges from 0.2% to 7.9%, and the prevalence and incidence of AF in western populations fluctuate between 0.5% to 7.5%. In an epidemiological survey conducted by Guo et al.^2^ in southwest China, the prevalence of AF has increased by 20 times in the past 11 years, while AF‐related stroke has increased by 13 times. About 15% of strokes are related to AF each year, and AF increases the risk of stroke by three to five times.^3^ Anticoagulant therapy has also been shown to reduce embolic events in patients with AF^4^ or mechanical valves,^5^ whether ischemic stroke or systemic vascular embolic events. In addition, the most common cause of valvular heart disease in underdeveloped and developing countries is rheumatic heart disease, compared with degenerative valve disease, which is the most common cause in developed countries. Patients who have undergone mechanical heart valve surgery must take warfarin and antiplatelet aggregation drugs for a long time. At present, new oral anticoagulants have not been approved for patients after mechanical heart valve operation.

After cerebral hemorrhage patients resume oral anticoagulation drugs, the incidence of hemorrhagic events increases, which is one of the main risks that need to be evaluated for anticoagulation. Hemorrhagic events especially in the elderly are often cited as an important reason to prevent the continuation of oral anticoagulants. At present, most of the risk assessments are combined with HAS‐BLED scores for bleeding assessment.^4^ For patients with a high bleeding risk with HAS‐BLED score >3, more accurate assessments are needed.

However, for patients with cerebral hemorrhage, it is still a topic to be discussed whether and when anticoagulation should be resumed. According to related studies, the risk of recurring ICH is 7% for warfarin (vitamin K antagonist [VKA]) and 5.07% for non‐VKA oral anticoagulants (NOAC).^6^ Restarting anticoagulation (RA) too early may increase the incidence of bleeding in patients. RA therapy too late leads to embolism, especially for patients who use mechanical heart valves. Therefore, there is still a lack of accurate answers to this question. In a systematic review and meta‐analysis of 3431 patients with various anticoagulant indications, Zhou et al.^7^ found that resumption of anticoagulation after ICH was not significantly associated with a higher risk of hemorrhagic events and long‐term mortality, but it was associated with a lower risk of ischemic events. However, no conclusion was reached as to when to resume anticoagulation therapy. Murthy et al.'s^8^ team found similar findings, and overall, all recommended RA) after ICH. However, they included all patients with ICH after admission, including those who died early in the hospital and did not restart anticoagulation. In addition, they included a controlled study of warfarin versus NOAC in their analysis. We believe that the inclusion of this group of patients could affect the final result, so we decided to further improve the analysis and find out the occurrence time of RA. Otherwise, we believe that the endpoint events including only ICH, ischemic stroke (IS), and long‐term mortality are lacking in feasibility. Because the probability of concurrent ICH in patients after RA is about 7%, not all concurrent ICH are fatal. However, for patients and families, any bleeding events can affect adherence to long‐term anticoagulation therapy. Therefore, we extended bleeding events to include all anticoagulation‐induced bleeding, including gastrointestinal bleeding, while the thrombotic events include arterial or venous thrombosis.

The European Society of Cardiology suggests that patients with AF can restart anticoagulation after 4–8 weeks when their condition is stable and under control.^9^ Most of the current observational studies have not yielded a definitive restart time, with most focusing on around 2 and 4 weeks. Related research has carried out a retrospective study on this issue, but it is difficult to provide strong evidence due to the sample size. We conducted a meta‐analysis of such studies to find out whether RA therapy after cerebral hemorrhage is effective and safe.

## 2 METHODS

We performed this study in accordance with guidelines recommended by the Preferred Reporting Items for Systemic Reviews and Meta‐Analyses (PRISMA) statement.^10^ We searched Medline, Embase, and the Cochrane Central Register of Controlled Trials from inception to November 2020. The data that was used in this study was publicly available, deidentified published, and exempt from approval by the ethics committee.

### 2.1 Data sources and searches

We searched in Medline, Embase, and the Cochrane Library from inception to November 2020. An English filter was used. Our research was based on the following medical subject headings terms or keywords: “intracranial hemorrhage or brain hemorrhage” and a combination of “anticoagulant,” “warfarin,” “atrial fibrillation,” “heart valve,” ‘ischemic stroke,” “brain Infarction,” “platelet aggregation inhibitors,” “myocardial infarction,” and “recurrence.”

### 2.2 Eligibility criteria

We summarize randomized controlled trials (RCTs) or cohort studies of patients receiving oral anticoagulants at the onset of ICH and survival in the acute phase or after hospitalization, evaluating long‐term mortality, thromboembolic complications, and intracerebral hemorrhage diagnosed in hospital Recurrent cerebral hemorrhage. The inclusion criteria for our study were: (1) adult patients ≥18 years of age; (2) studies with nontraumatic ICH; (3) sample size ≥18 patients to avoid small case report; (4) studies with outcomes of myocardial infarction (MI), IS, and recurrence ICH in the follow‐up period; (5) studies with a clear statement of whether restart AC. We excluded the following studies: (1) included mixed populations (such as AC and antiplatelet aggregation) and failure to individually separate results of the patients with ICH; (2) outcomes were not reported separately for participants resume anticoagulant therapy (or switched to antiplatelet drugs); (3) case report, review, guidelines, and book chapters (not provide details of data).

### 2.3 Study selection and quality assessment

A single researcher read the title and abstract from the initial search and made a short list for review in full text. Each full‐text article was reviewed by two independent researchers based on the inclusion criteria, exclusion criteria, and quality of data. Any disagreements on data were resolved by a third investigator. Use prespecified collection templates to extract data. Two research also judged the quality of each article according to the Jadad scale^11^ for assessing the risk of bias.

### 2.4 Outcomes

The outcomes of interest were acute and long‐term mortality after hospitalization, hemorrhagic events (including ICH and gastrointestinal bleeding), and ischemic events (including deep vein thromboembolism, MI, pulmonary embolism, IS, and systemic embolism). The following characteristics were extracted: journal of publication, the authors, year of publication, country of the study, and the follow‐up time. We collected patient‐related information, including age, sex, and stroke comorbidities, such as diabetes mellitus, hypertension, coronary artery disease, smoking history, mechanical heart valve, atrial fibrillation, prior stroke, prior transient ischemic attack, heart valve replacement, time of restarting AC, and heart failure.

### 2.5 Data analysis

We performed a meta‐analysis to assess the association between the no anticoagulation group and AC resumption and endpoint events by using the pooled odds ratio (OR). OR with a 95% confidence interval (CI) was estimated by using a random‐effects (DerSimonian and Laird^12^) model. We generated forest plots to display the individual study OR and pooled OR. Statistical analyses were performed using Review Manager 5.0. In every case, a two‐tailed *p* < 0.05 was considered significant.

## 3 RESULTS

### 3.1 Study selection and characteristics

**Figure 1:**
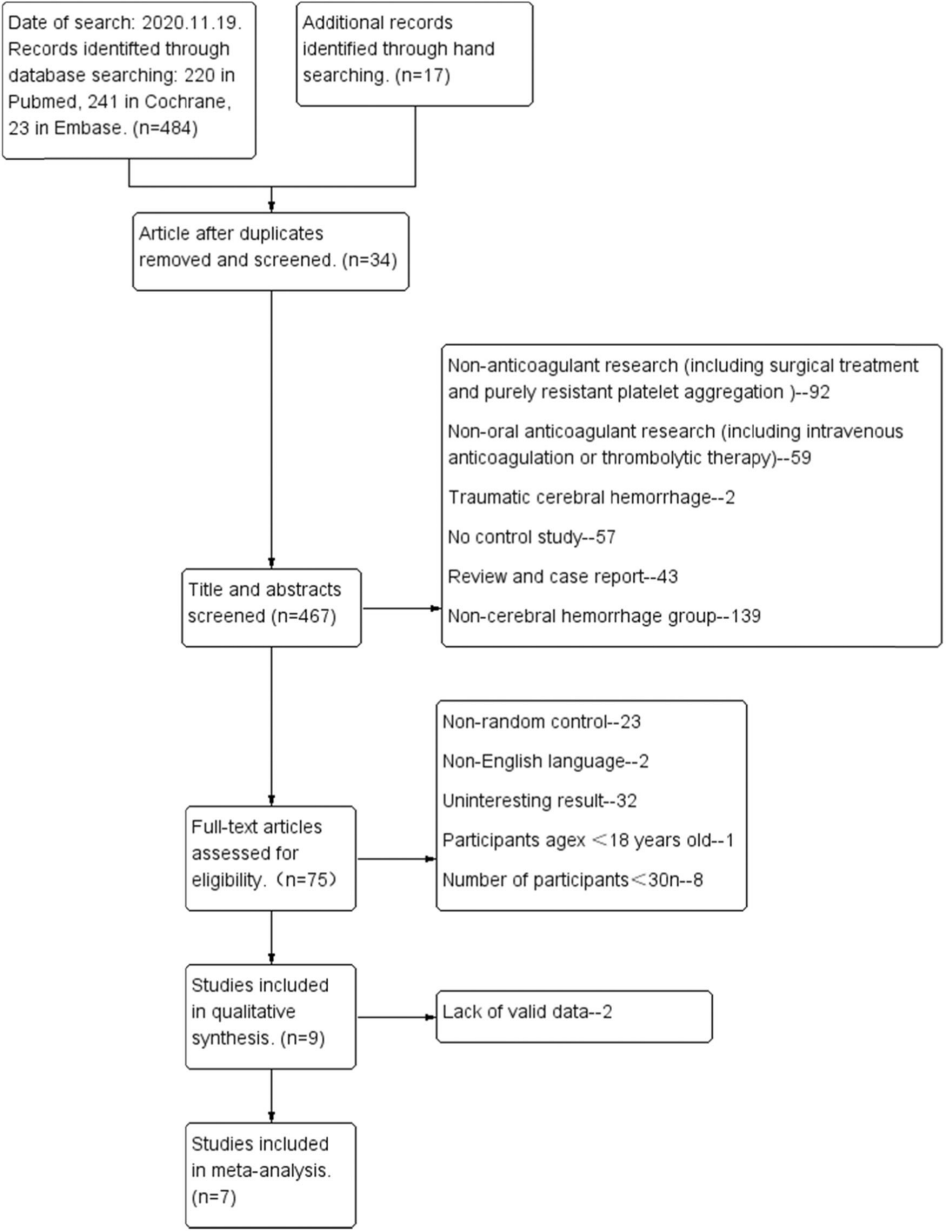
Flow chart of literature search. The search yielded 501 papers, with 34 duplicates removed. After reading the titles and abstracts 392 were removed, including reviews, case reports, lack of control group studies, traumatic brain hemorrhage, lack of studies of oral anticoagulants, and so forth. Read the full article to remove 66, including studies with participants younger than 18 years, studies with sample sizes of less than 30, and studies with uninteresting endpoint events. Finally, seven studies were included after removing two studies that lacked valid data.

We screened a total of 501 titles and abstracts, of which 75 articles were reviewed in full text (Figure 1). For the evaluation of thromboembolic events after ICH, seven studies (no RCTs, seven retrospective cohorts) of 1876 participants (664 resume anticoagulant therapy, 1212 do not resume anticoagulant therapy) met the inclusion criteria. There were 198 events with ICH recurrence, 175 events with thromboembolic events, and 251 died during the follow‐up time (Table 1). We considered surviving patients to be more valuable for the study and collected patients who were still alive during the restarted anticoagulation time interval (patients who died within a short period after admission were excluded). The total number of selected studies, included two from the United States,^13^, ^14^ two from Canada,^15^ one from Canada and Sweden,^16^ one from Belgium,^17^ one from Germany,^18^ and one from Korea.^19^ After statistics, for the selection of patients with intracranial hemorrhage, two studies defined hemorrhage in the intracranial brain parenchyma,^13^, ^18^ while five studies expanded the inclusion criteria for cerebral hemorrhage to include subarachnoid hemorrhage, subdural hematoma (Table 2).^14^, ^15^, ^16^, ^17^, ^19^

**Table 1:** Outcomes of included studies

Study	Anticoagulant (*n*)				No anticoagulant (*n*)			
	Hemorrhage events	Thromboembolic events	Death	Total	Hemorrhage events	Thromboembolic events	Death	Total
de Vleeschouwer et al. (2005)^17^	1	0	NA	25	7	3	NA	81
Claassen et al. (2008)^13^	3	6	3	23	2	13	8	25
Majeed et al. (2010)^16^	8	2	NA	45	10	19	NA	87
Yung et al. (2012)^15^	14	NA	44	91	29	NA	118	193
Witt et al. (2015)^14^	5	2	10	54	10	13	33	105
Kuramatsu et al. (2015)^18^	14	9	NA	172	36	82	NA	547
Park et al. (2016)^19^	46	9	13	254	13	17	22	174
Total	91	28	70	664	107	147	181	1212

Abbreviation: NA, not applicable.

**Figure 2:**
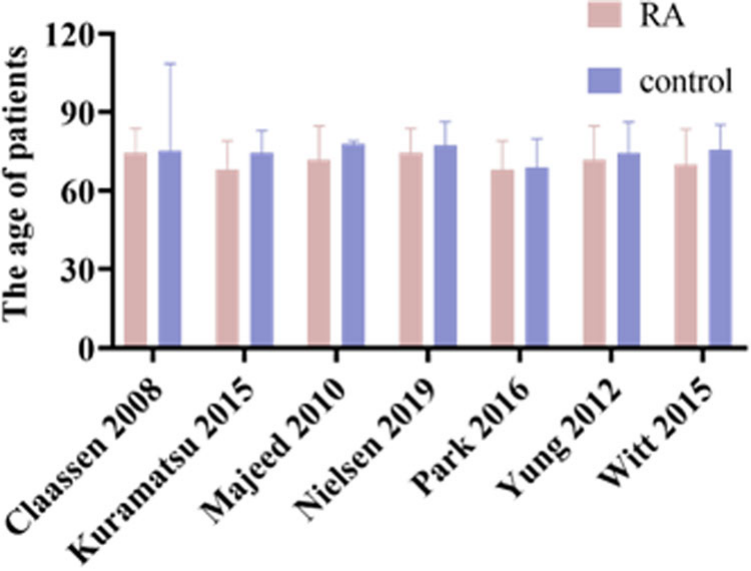
The mean age comparison between the restarted anticoagulation and control groups (RA = restarting anticoagulation, control = not restarting anticoagulation, data expressed as mean ± standard deviation) [Color figure can be viewed at wileyonlinelibrary.com]

The mean age of the RA group ranged from 67–71 years old and the mean age of the control group ranged from 69–76 years old (Figure 2). Males made up the majority of the patient composition (Table 2). In those with an indication for anticoagulation, the most common comorbidity is AF, followed by a history of prior stroke and prosthetic heart valve disease (Table 3).

**Table 2:** Characteristics of included studies

Study	OAC type	Male (total)	Mean age (years)	Received OAC (*n*)	Time to RA	Follow‐up
de Vleeschouwer et al. (2005)^17^	VKA	65 (106)	NA	25	11 days	7 years
Claassen et al. (2008)^13^	Warfarin	27 (48)	70.5	23	<60 days	49.8 months
Majeed et al. (2010)^16^	Warfarin	87 (132)	76	45	4.4 weeks	34 months
Yung et al. (2012)^15^	Warfarin	156 (284)	73.5	91	<1 month	1 year
Witt et al. (2015)^14^	Warfarin	84 (160)	73.6	54	<2 weeks	1 year
Kuramatsu et al. (2015)^18^	Warfarin	442 (719)	74.1	172	1 month	1 year
Park et al. (2016)^19^	VKA	146 (428)	68.3	254	117.5 days	3.5 years

Abbreviations: NA, not applicable; OAC, oral anticoagulants; RA, restarting anticoagulation; VKA, vitamin K antagonist.

**Table 3:** Baseline of included studies

Study	Indications for anticoagulation (*n*)					Country	Design
	Prosthetic heart valve	Prior stroke	MI	VTE	NVAF		
de Vleeschouwer et al. (2005)^17^	30	17	2	4	56	Belgium	RC
Claassen et al. (2008)^13^	12	15	10	10	23	USA	RC
Majeed et al. (2010)^16^	22	59	NA	0	22	Canada & Sweden	RC
Yung et al. (2012)^15^	37	67	NA	31	19	Canada	RC
Witt et al. (2015)^14^	23	15	NA	45	49	USA	RC
Kuramatsu et al. (2015)^18^	50	NA	NA	31	566	Germany	RC
Park et al. (2016)^19^	79	154	NA	26	428	Korea	RC
Total	253	327	12	147	1163		

Abbreviations: MI, myocardial infarction; NA, not applicable; NVAF, nonvalvular atrial fibrillation; RC, retrospective cohort; VTE, vein thromboembolism.

### 3.2 Meta‐analysis of resuming anticoagulant therapy with outcomes

#### 3.2.1 Risk of bias

**Figure 3:**
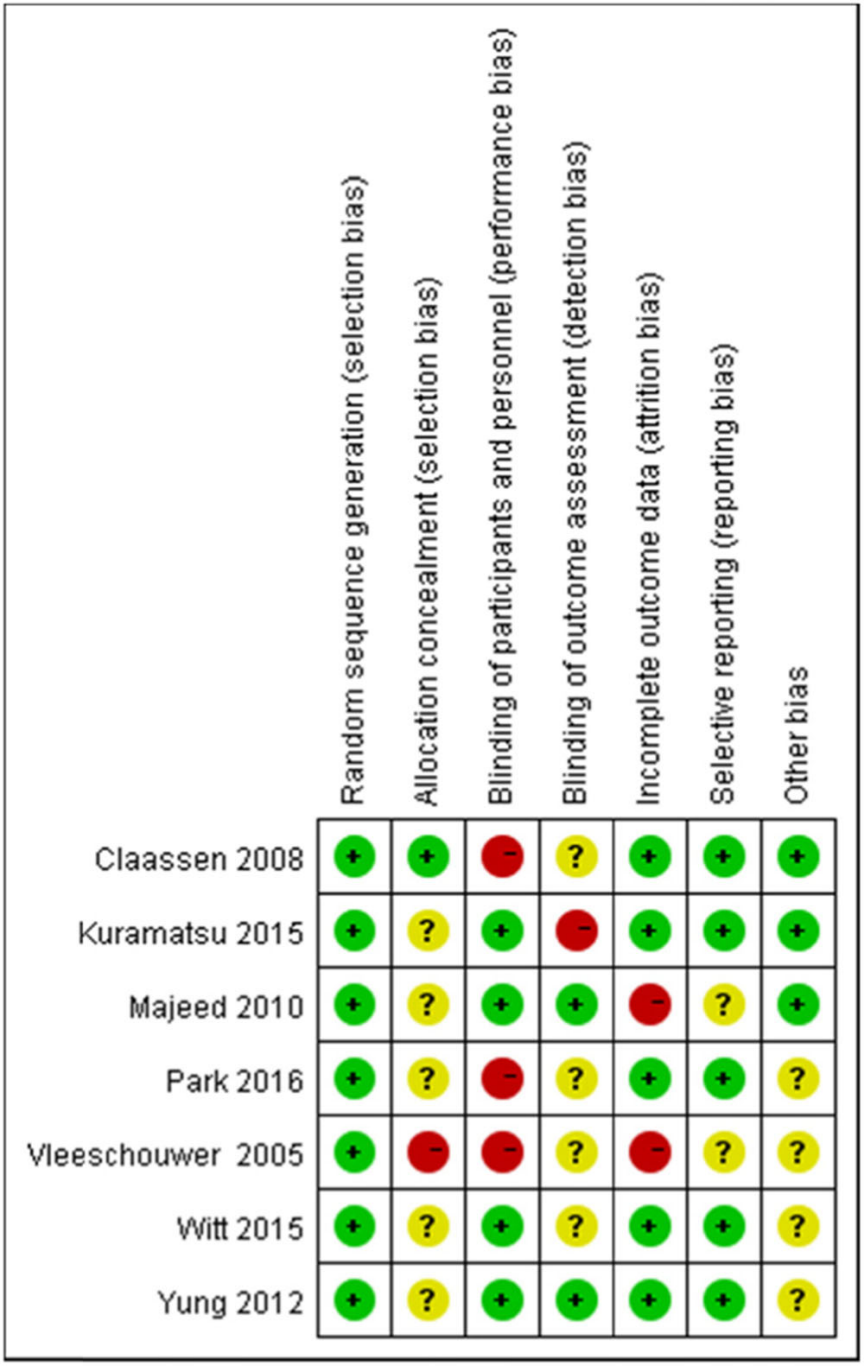
The risk of bias is assessed for selection bias, performance bias, detection bias, attrition bias, reporting bias, and other biases (Green means low risk of bias. Yellow means unclear risk of bias. Red means high risk of bias). VTE, vein thromboembolism [Color figure can be viewed at wileyonlinelibrary.com]

**Figure 4:**
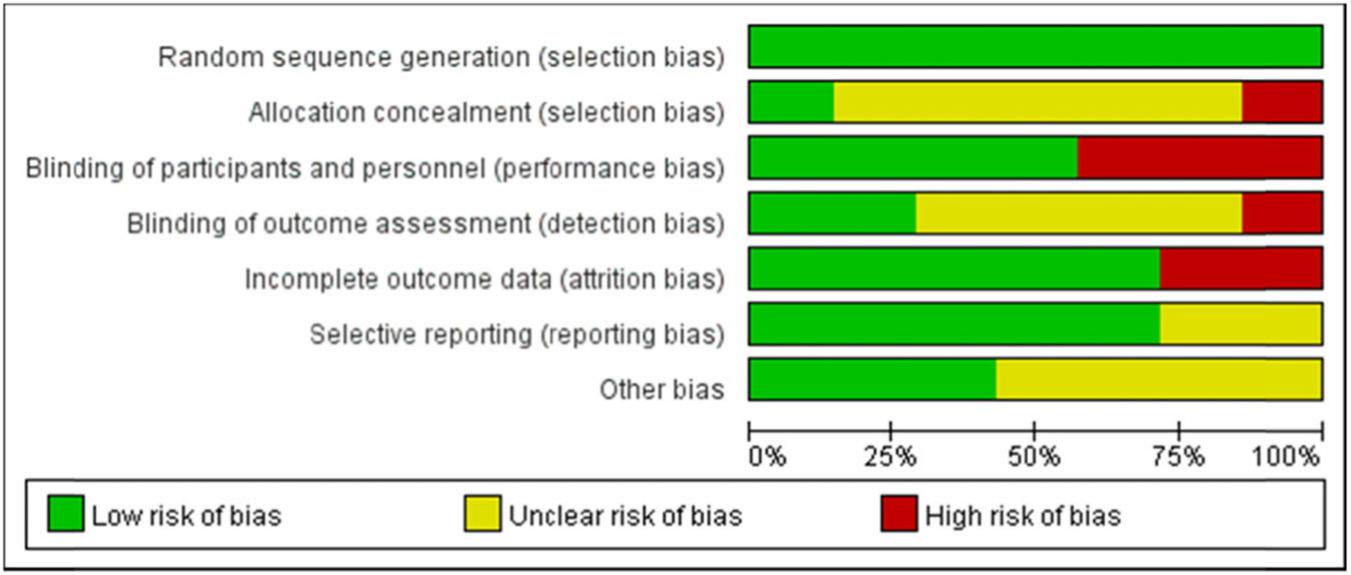
Percentage of the combined risk of bias assessment for included studies. Among the included studies, the risk of selection bias, reporting bias, and other biases are lower. (Green means low risk of bias. Yellow means unclear risk of bias. Red means high risk of bias). [Color figure can be viewed at wileyonlinelibrary.com]

In short, most of the risk of blinding comes from an unspecified risk of selective blinding. This is mainly due to incomplete data reporting, selective presentation of results and other bias (Figures 3 and 4).

### 3.3 Relationship between restarting oral anticoagulation with hemorrhagic events

**Figure 5:**
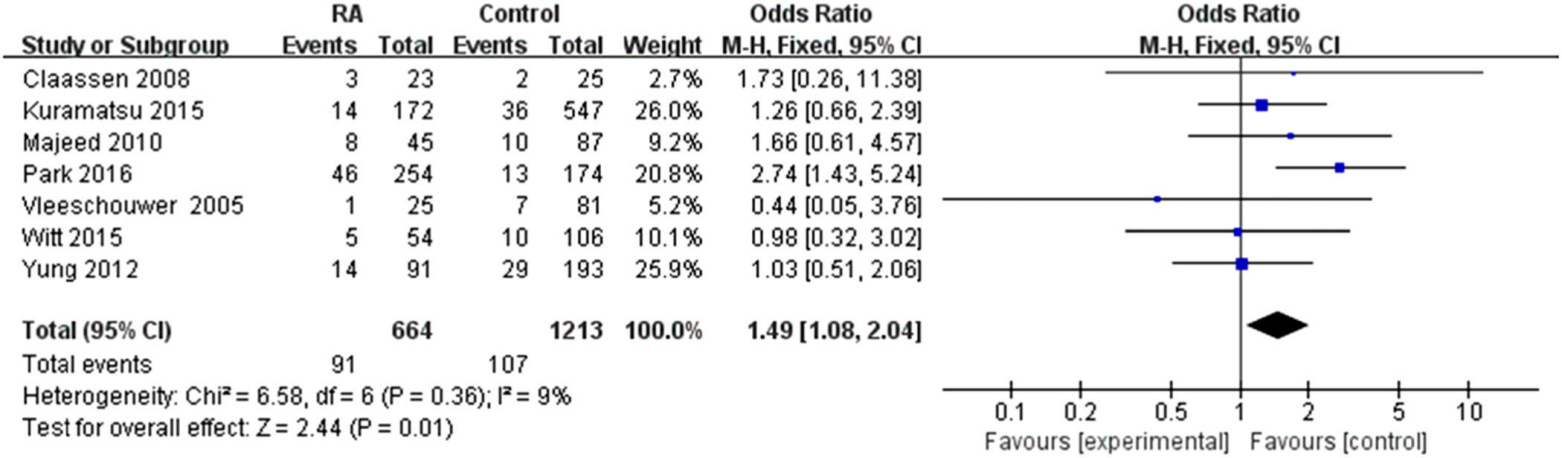
Forest plot of the relationship between the resumption of oral anticoagulation therapy after intracranial hemorrhage and complications of hemorrhagic events. Aggregated relative risks are shown in the forest plot. Each square represents a point estimate of the effect size for any given study, and the horizontal line represents the 95% confidence interval (CI) for each study. The diamonds represent the pooled estimates, and the width of the diamond represents the pooled 95% CI. Pooled odds ratio 1.49, 95% CI 1.08–2.04, *p* = 0.01. [Color figure can be viewed at wileyonlinelibrary.com]

A meta‐analysis of seven studies is consistent with the relationship between RA therapy and the occurrence of hemorrhagic events. These contained a total of 1877 intracranial hemorrhagepatients, and anticoagulation was restarted in 664 patients (35.3%). Hemorrhagic events occurred in 91 (13.7%) patients who received anticoagulation and 107 (8.8%) patients who did not receive antithrombotic therapy. It was an increased incidence of hemorrhagic events in the RA group compared to the control group (pooled OR 1.49, 95% CI 1.08*–*2.04, *p* = 0.01). Heterogeneity was less in the restarted anticoagulation treatment group compared to the control group (*I* ^2^ = 9%, *p* = 0.36) (Figure 5). Resumption of anticoagulation can lead to increased hemorrhagic events.

### 3.4 Relationship between restarting oral anticoagulation with ischemic events

**Figure 6:**
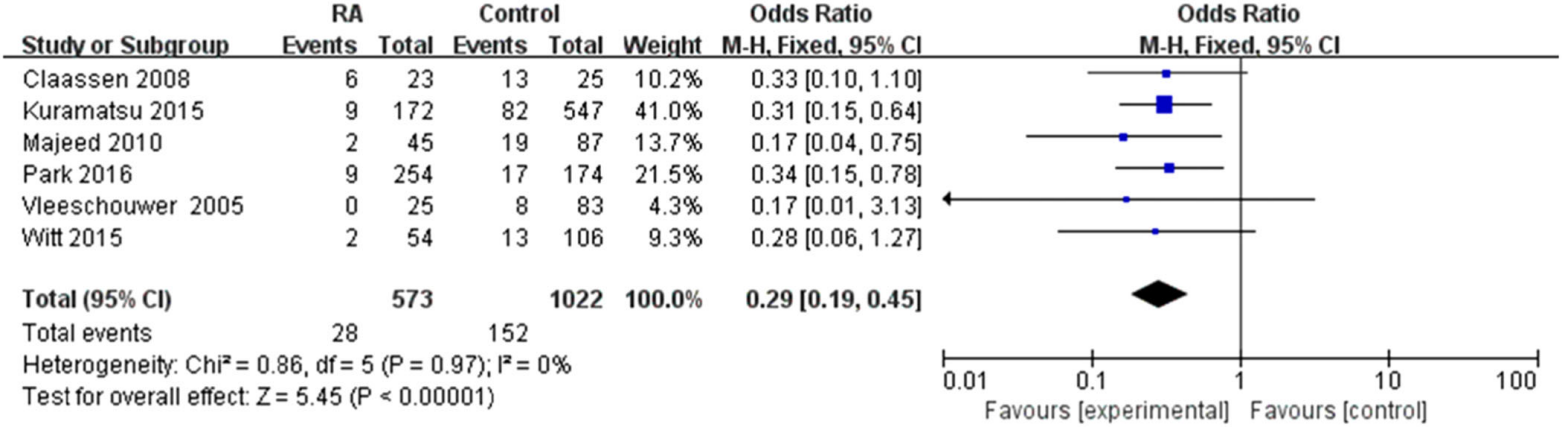
Forest plot of the relationship between the resumption of oral anticoagulation therapy after intracranial hemorrhage and complications of thromboembolic events. The meta‐analysis was calculated using a random‐effects model, with the pooled relative risk shown in the forest plot. Pooled odds ratio 0.29, 95% confidence interval (CI) 0.19–0.45, *p* < 0.00001. [Color figure can be viewed at wileyonlinelibrary.com]

Six studies reported the occurrence of ischemic events between restarting oral anticoagulation with control groups. This contained a total of 1595 intracranial hemorrhage patients, while 573 patients (35.9%) were restarting anticoagulation. Ischemic events occurred in 28 (4.8%) patients who received anticoagulation and 152 (14.8%) patients who did not receive antithrombotic therapy. There was a decreased incidence of ischemic events in the restarted anticoagulation group compared to the control group (pooled OR 0.29, 95% CI 0.19–0.45, *p* < 0.00001). Compared with the control group was statistically significant with no evidence of significant heterogeneity (*I* ^2^ = 0%, *p* = 0.97) (Figure 6). Resumption of anticoagulation therapy can lead to a reduction in embolic events.

### 3.5 Relationship between restarting oral anticoagulation with death events

**Figure 7:**
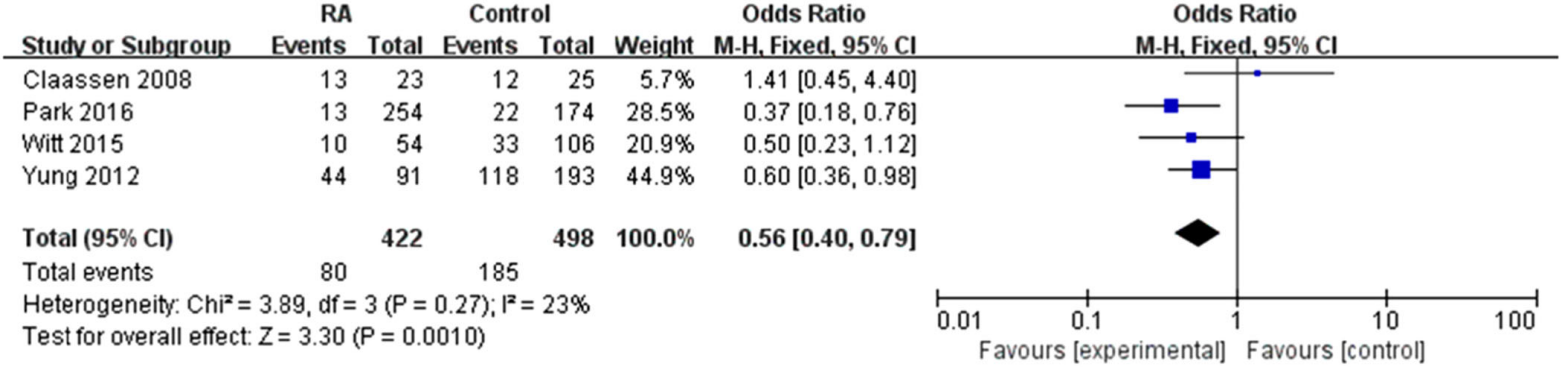
Forest plot of the relationship between the resumption of oral anticoagulation therapy after intracranial hemorrhage and the endpoint event of death. The meta‐analysis was calculated using a random effects model, with the pooled relative risk shown in the forest plot. Pooled odds ratio 0.56, 95% confidence interval (CI) 0.40–0.79, *p* = 0.001. [Color figure can be viewed at wileyonlinelibrary.com]

Six studies reported the occurrence of death events between restarting oral anticoagulation with control groups. This contained a total of 920 intracranial hemorrhage patients, while 422 patients (45.8%) were restarting anticoagulation. Death events occurred in 80 (18.0%) patients who received anticoagulation and 185 (37.1%) patients who did not receive antithrombotic therapy. There was a decreased incidence of death events in the restarted anticoagulation group compared to the control group (pooled OR 0.56, 95% CI 0.40–0.79, *p* = 0.001). Compared with the control group was statistically significant with no evidence of significant heterogeneity (*I* ^2^ = 23%, *p* = 0.27) (Figure 7). Resumption of anticoagulation reduces long‐term mortality.

### 3.6 Optimal time to restart anticoagulation

**Figure 8:**
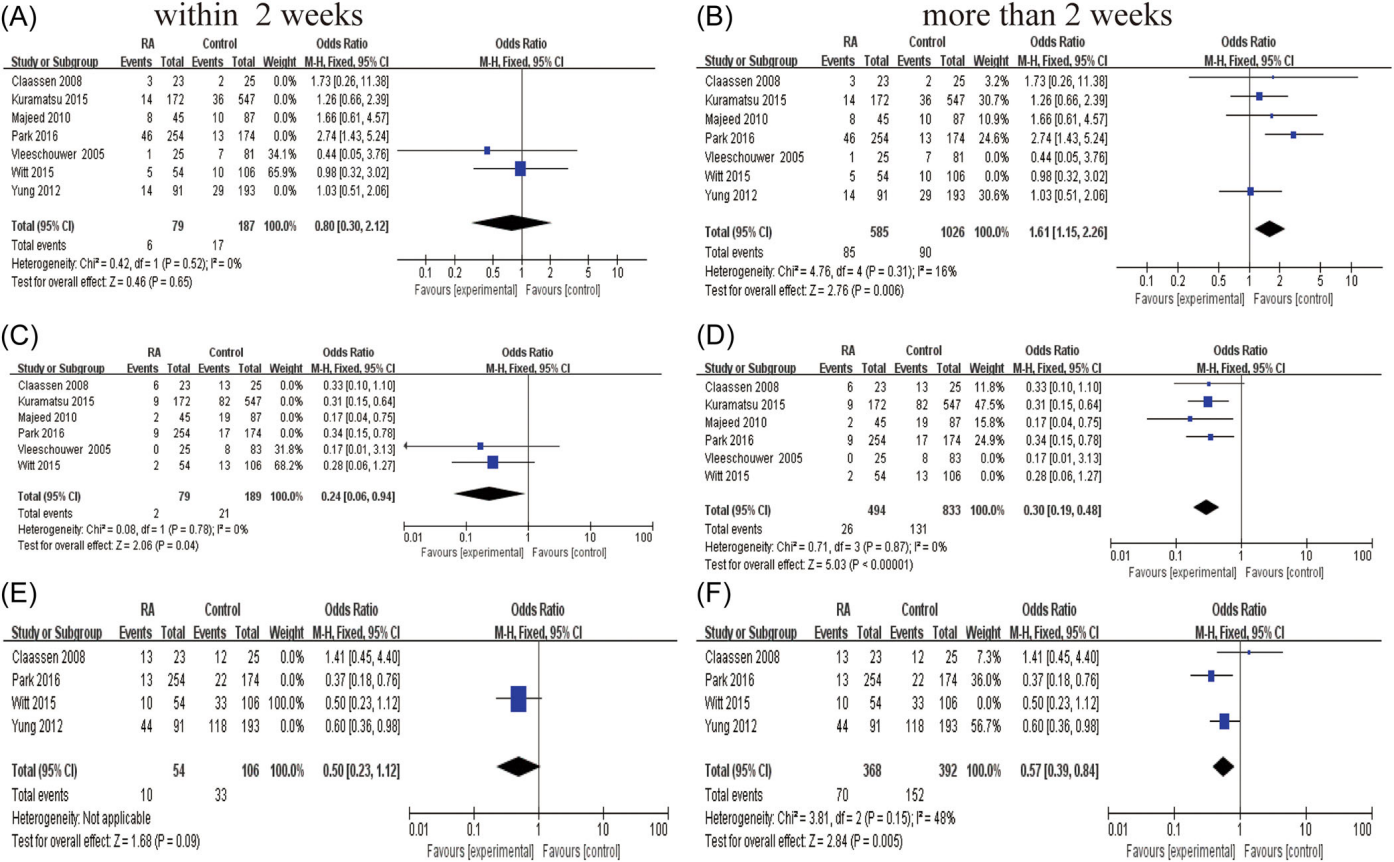
Time to restart anticoagulant therapy for intracranial hemorrhage: a forest plot of the relationship between endpoint events within and after 2 weeks. (A, B) Hemorrhagic events. (C, D) Ischemic events. (E, F) Death events. (A, C, E) Restarting anticoagulant therapy within 2 weeks. (B, D, F) Restarting anticoagulant therapy after 2 weeks. [Color figure can be viewed at wileyonlinelibrary.com]

The incidence of bleeding events was analyzed by dividing into two major groups according to the time of RA. We found no significant increase in the incidence of hemorrhagic events in patients who restarted anticoagulation within 2 weeks, compared with the group who restarted anticoagulation after 2 weeks (pooled OR 0.80, 95% CI 0.3–2.12, *p* = 0.65 vs. OR 1.61, 95% CI 1.15–2.26, *p* = 0.006) (Figure 8A,B). Compared with the control group was statistically significant with no evidence of significant heterogeneity (within 2 weeks, *I* ^2^ = 0, *p* = 0.52; more than 2 weeks, *I* ^2^ = 16%, *p* = 0.31). In other words, RA therapy within 2 weeks does not increase the patient's risk of hemorrhagic events. There was no significant change in the incidence of endpoint events associated with ischemic events and death within 2 weeks of RA versus after 2 weeks (Figure 8C–F).

**Figure 9:**
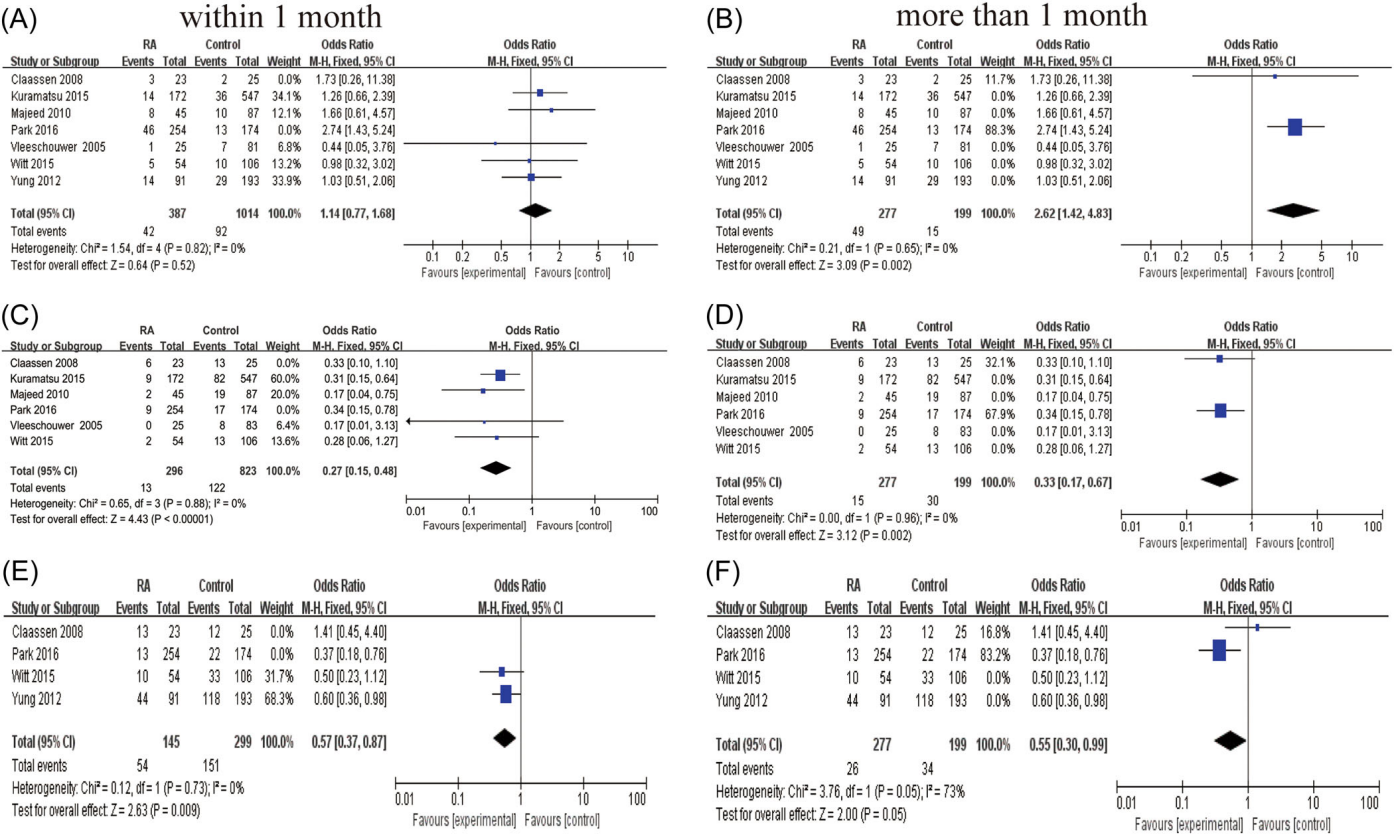
Time to restart anticoagulant therapy for intracranial hemorrhage: a forest plot of the relationship between endpoint events within and after 1 month. (A, B) Hemorrhagic events. (C, D) Ischemic events. (E, F) Death events. (A, C, E) Restarting anticoagulant therapy within 1 month. (B, D, F) Restarting anticoagulant therapy after 1 month. [Color figure can be viewed at wileyonlinelibrary.com]

There was no significant correlation between the occurrence of bleeding events and whether anticoagulation was restarted in patients whose anticoagulation was restarted within 1 month after cerebral hemorrhage, and no significant increase in the incidence of hemorrhagic events was seen in patients whose anticoagulation was restarted after 1 month (pooled OR 1.14, 95% CI 0.77–1.68, *p* = 0.52 vs. OR 2.62, 95% CI 1.42–4.83, *p* = 0.002) (Figure 9A,B). There was no significant change in the incidence of endpoint events associated with ischemic events and death within 2 weeks of RA versus after 2 weeks (Figure 9C–F).

## 4 DISCUSSION

Through a meta‐analysis of seven retrospective studies, we found that resumption of anticoagulation therapy was associated with a lower risk of ischemic and death events. In addition, resumption of oral anticoagulation within 2 weeks or 1 month did not significantly increase the risk of hemorrhagic events.

### 4.1 Effectiveness of anticoagulation

In current clinical practice, there is a gradual increase in the number of patients taking oral anticoagulants for the prevention of ischemic events. In relevant epidemiological investigations, Asians are more likely to bleed from oral warfarin than non‐Asians.^20^, ^21^, ^22^ This also makes the use of warfarin, especially in Asia, more difficult. Moreover, oral anticoagulation therapy is required to maintain the international normalized ratio (INR) in the therapeutic range. The risks of bleeding events and embolism with VKA anticoagulants can be predicted by time in the therapeutic range (TTR). According to the guidelines, TTR control of 70% and an INR between 2.0 and 3.0 are recommended to prevent stroke and minimize the risk of bleeding,^23^ especially in AF.^24^ In our meta‐analysis, we found a reduced risk of ischemic events, including stroke, and a slight increase in major bleeding events in the oral VKA group compared to the control group.

Inaccurate control of TTR is an important cause of increased incidence of bleeding events or ischemic events in patients. It also, therefore, restricts the use of VKA in clinical practice, in contrast to new oral anticoagulants that do not require coagulation monitoring. Moreover, literature has indicated that some patients with heart valve disease require short‐term co‐administration of an antiplatelet aggregation inhibitors in conjunction with oral VKA, which is an important reason for the treatment of cerebral hemorrhage that is prone to recurrence.^21^, ^25^ Therefore, the key to the effectiveness of oral anticoagulants is how to stabilize the INR as well as the TTR within the range of efficacy. This will reduce the occurrence of not only bleeding events but also thrombotic events. When this target cannot be met for social, economic, or transportation reasons, we may need to choose new oral anticoagulants or antiplatelet aggregation drugs to ensure that patients can benefit from them.

### 4.2 Anticoagulant therapy or antiplatelet therapy

In individual studies, the resumption of antiplatelet therapy appears to be safer than the resumption of anticoagulant drugs. However, due to patient disease factors, such as patients with atrial fibrillation, or after valve replacement in rheumatic heart disease, anticoagulation is a better and preferable option in preventing thrombosis. Therefore, oral medication cannot be defined or recommended for patients purely in terms of safety.

### 4.3 Time of restart anticoagulation

There are still no clear guidelines on the best time to resume anticoagulation in patients with cerebral hemorrhage. Studies related to RA after cerebral hemorrhage include those of Apixaban versus Antiplatelet drugs or no antithrombotic drugs after anticoagulation‐associated intraCerebralHaEmorrhage in patients with Atrial Fibrillation (APACHE‐AF), the REstart or STopAntithromboticsRandomised Trial (RESTART).^26^ Most studies currently recommend RA at >4 weeks.^27^, ^28^

In addition, we selected the participants who survived the acute phase or hospitalization to avoid the impact of patients who die early due to excessive disease. We consider these overly critical patients to influence the endpoint events of RA, as the long‐term outcome is the key factor influencing clinical decisions. This systematic review and meta‐analysis showed that RA after ICH within 2 weeks^14^, ^17^ reduced the incidence of ischemic events without increasing the risk of death events and hemorrhagic events. Most of the studies restarted anticoagulation within 1 month and had the same advantages.^14^, ^15^, ^16^, ^17^, ^18^

### 4.4 Type of hemorrhage

In addition to the timing of RA, the site of intracranial hemorrhage (deep or lobar) seems to be an issue worth considering. Two studies included only parenchymal hemorrhage,^13^, ^18^ while the remaining five studies expanded the inclusion criteria to include subarachnoid hemorrhage, subdural hemorrhage, and so forth.^14^, ^15^, ^16^, ^17^, ^19^ Subarachnoid hemorrhage due to aneurysm rupture is associated with reduced recurrent intracranial hemorrhage events with oral anticoagulants after aneurysm occlusion because of its clear etiology.^29^ Thus expanding the inclusion criteria may lead to a reduction in bleeding events after oral anticoagulants and to a false negative conclusion. This is one of the reasons why it is currently difficult to obtain a uniform restart anticoagulation time.

### 4.5 Study limitations

First, all the studies we included were retrospective and had the disadvantage of being nonblinded. In clinical practice, restarting oral anticoagulants, especially after cerebral hemorrhage, must be informed of the benefits and risks and requires a signed written consent. Therefore, it is difficult to conduct randomized, double‐blind research studies. Second, the clinical heterogeneity of patient composition is large because of the multifactorial etiology of cerebral hemorrhage, which is predominantly in the elderly. Thus, age, education, economic factors, social factors, and comorbidities, including diabetes, hypertension, and renal impairment, lead to a high degree of heterogeneity in each patient, and differences in baseline can lead to limitations in the subsequent outcome. This is the reason why the results obtained so far have been difficult to generalize on a large scale. In addition to this, some of the studies included patients with subarachnoid hemorrhage due to aneurysm rupture as well as patients with subdural hemorrhage. Patients with subarachnoid hemorrhage due to aneurysm will have a lower risk of bleeding after RA than patients with lobar hemorrhage because of the clarity of the etiology.^30^

Second, despite the inclusion of a large total sample, the low incidence of each event also limited us to conduct further multifactorial and subgroup analyses. There were not enough samples to explore confounding factors, and therefore the relevant results were also vulnerable to confounding factors, such as indications and inclusion conditions. In addition, some of the missing data can affect the final result of the article.

## 5 CONCLUSION

Based on these retrospective cohorts, recovery of anticoagulation after ICH is beneficial for long‐term mortality and recurrence of ischemic events. The meta‐analysis showed that resumption of oral anticoagulation within 2 weeks or 1 month in patients who had a cerebral hemorrhage was beneficial and did not increase the risk of hemorrhagic events and reduced the occurrence of ischemic events and fatal endpoint events.

## AUTHOR CONTRIBUTIONS

Xue‐Yan Huang and Jun‐Yan Zhang conceived the idea; Xue‐Yan Huang conducted the analyses; Jun‐Yan Zhang and Chang‐Yin Yu provided the data; all authors contributed to the writing and revisions.

## CONFLICT OF INTEREST

The authors declare no conflict of interest.

## ETHICAL STATEMENT

Not applicable.

## TRANSPARENCY STATEMENT

All the authors affirm that this manuscript is an honest, accurate, and transparent account of the study being reported. No important aspects of the study have been omitted and that any discrepancies from the study as planned (and, if relevant, registered) have been explained.

## Data Availability

The data sets generated during and/or analyzed during the current study are available from the corresponding author on reasonable request.
